# Eine unerwartete Entdeckung bei einer Patientin mit chronischer Prurigo

**DOI:** 10.1007/s00105-023-05131-8

**Published:** 2023-03-13

**Authors:** Elisabeth Steffens, Mustafa Kaplan, Elke Weisshaar

**Affiliations:** Sektion Berufsdermatologie, Hautklinik, Universitätsklinikum Heidelberg, Voßstraße 2, 69115 Heidelberg, Deutschland

**Keywords:** Karzinom, Chronische Prurigo, Jucken, Prurigo nodularis, Pruritus, Cancer, Itch, Chronic prurigo, Prurigo nodularis, Pruritus

## Abstract

Wir berichten über eine 61-jährige Patientin, die seit 1 Jahr an plötzlich aufgetretenem, starkem Pruritus und Knoten auf der Haut des gesamten Integuments litt. Es wurde die Diagnose einer chronischen Prurigo (CPG) gestellt. Im Rahmen einer umfassenden und interdisziplinären Durchuntersuchung wurde ein metastasiertes Ovarialkarzinom festgestellt. Nach radikaler operativer Therapie und Chemotherapie heilte die CPG ab und ist bis heute nicht rezidiviert. Es ist nicht auszuschließen, dass es sich hier um eine paraneoplastische CPG handelt. Dieser Fall demonstriert auch, dass bei CPG eine Ursache gefunden werden kann und daher eine gründliche Abklärung einer CPG sehr wichtig ist und auch lebensrettend sein kann.

## Abstract (en)

We report on a 61-year-old woman with a sudden onset of itching and nodules on the skin for the last year. Chronic prurigo (CPG) was diagnosed. An extensive and interdisciplinary check-up revealed metastasized ovarian cancer. Radical surgery and chemotherapy followed. The CPG has completely healed and has not relapse. We believe this case to represent paraneoplastic CPG. This case report also demonstrates that the etiology of CPG can be identified and that a detailed workup is worthwhile and can be life-saving.

## Anamnese

Eine zuvor hautgesunde, 61-jährige Patientin berichtete über plötzlich aufgetretenes Jucken, welches seit 1 Jahr bestand und mit einer ausgeprägten Knotenbildung der Haut einherging. Es waren mehrere ambulante Vorstellungen bei Dermatologen und ein stationärer Aufenthalt erfolgt, bei denen verschiedene topische Therapien, systemische Therapien mit Antihistaminika und UV-Phototherapie keine Linderung erbracht hatten. An Vorerkrankungen waren eine pulmonale arterielle Hypertonie (seit Mai 2021 mit Bosentan, 62,5 mg täglich behandelt) und ein Raynaud-Syndrom (seit etwa Mitte 2021 mit Hydroxychloroquin, 200 mg täglich behandelt) bekannt. Die in den letzten 2 Jahren routinemäßig erfolgten gastroenterologischen sowie gynäkologischen Untersuchungen seien laut Angabe der Patientin unauffällig gewesen. Die Erstvorstellung in unserer Hautklinik erfolgte am 17.08.2021.

## Klinischer und histopathologischer Befund

**Figure 1:**
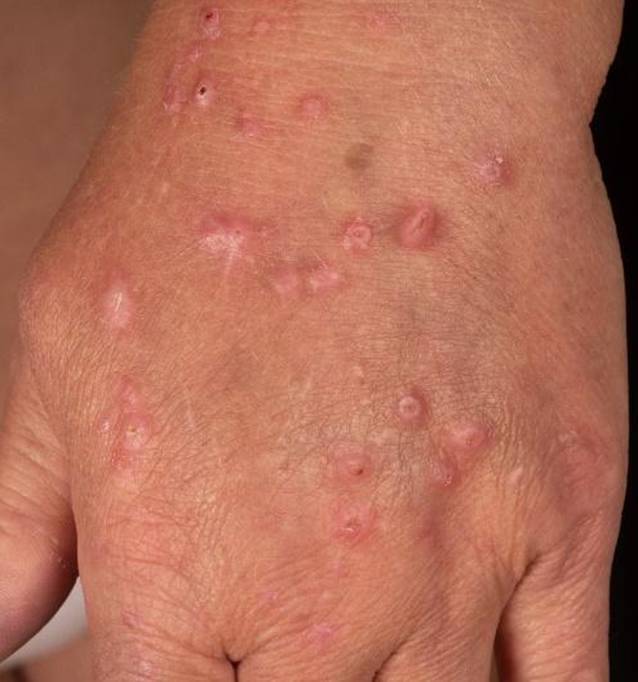

Die klinische Untersuchung ergab keinen Hinweis auf eine atopische Dermatitis (AD) oder eine andere Dermatose, es bestand auch keine atopische Hautdiathese. Das gesamte Integument, insbesondere der Rücken mit Aussparung interskapulär (sog. Schmetterlingszeichen = „butterfly sign“) und die Streckseiten der Arme, zeigten multiple Papeln und teilweise längsovale, teilweise runde Knoten, zum Teil mit krustig belegten Exkoriationen. Sowohl die histopathologische Untersuchung als auch die direkte Immunfluoreszenz einer am linken Unterarm entnommenen Hautbiopsie waren unauffällig. Es wurde eine chronische noduläre Prurigo diagnostiziert (Abb. 1).

## Diagnostik

Die leitliniengemäße Laboruntersuchung [3] war bis auf eine LDH-Erhöhung (301 U/l) unauffällig. Bei einer Sonographie des Abdomens zeigte sich eine 43 × 42 mm messende Raumforderung am Corpus uteri mit Verdacht auf Uterusmyom. Die Intensität des Pruritus wurde auf numerischen Rating Skala (NRS; 0–10) mit 9 angegeben.

## Therapie und Verlauf

**Figure 2:**
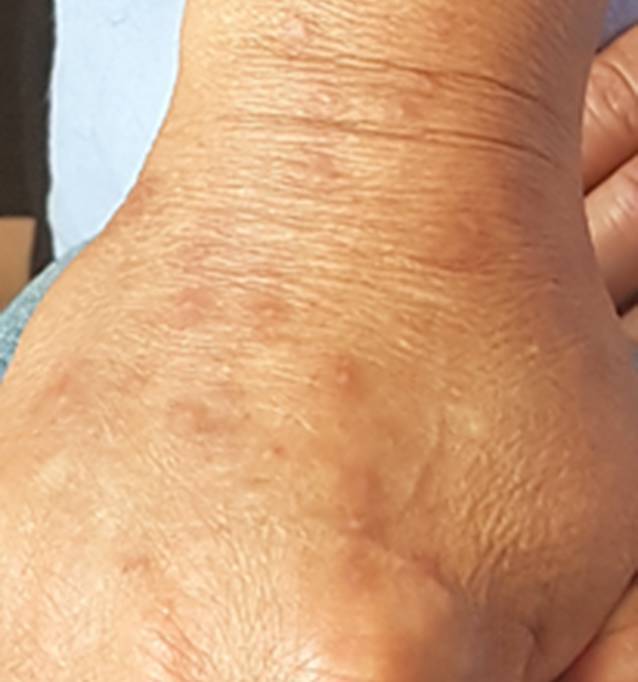

Da die Lokaltherapie mit Kortikosteroiden, Harnstoff, Ichthyol, auch in Kombination mit einer selektiven UV-Phototherapie, keine Besserung der CPG erbrachte, erfolgten der Einschluss und die Randomisierung in die sog. PRISM-Studie (Nalbuphin oral vs. Placebo). In der vom Radiologen zur weiteren Abklärung empfohlenen MRT-Untersuchung des Abdomens bestätigte sich die Raumforderung, die jetzt nicht mehr dem Uterus zugeordnet werden konnte. Die radikale operative Therapie einschließlich Hysterektomie, Ovarektomie, Lymphadenektomie, Entfernung des Peritoneums im rechten Oberbauch und der Rektumvorderwand, Cholezystektomie sowie Zwerchfellteilresektion mit histologischer Aufarbeitung bestätigte die Diagnose eines serösen High-grade-Ovarialkarzinoms (rechts und links), Metastasen an Rektum, Leber, Gallenblase, Peritoneum und Zwerchfell. Die Studienteilnahme musste nach 4 Studienvisiten beendet werden. Die adjuvante Chemotherapie erfolgte in 6 Zyklen mit je Paclitaxel 175 mg/m^2^, Carboplatin AUC 5 q3w, Dexamethason 16 mg und Bevacizumab 15 mg/kg q3w. Etwa 2 Monate nach Ende der Chemotherapie wurde kein Pruritus mehr beklagt (NRS = 0), und die CPG war abgeheilt (Abb. 2).

## Diskussion

Die CPG (zuvor als Prurigo nodularis bezeichnet) [2] ist eine chronische Dermatose, die verschiedene Ursachen haben kann, in vielen Fällen bleibt die zugrunde liegende Ätiologie jedoch ungeklärt [1, 3]. Die Ursachenfindung bei CPG ist bekanntermaßen eine Herausforderung und erfordert ein systematisches Vorgehen wie dies die aktuelle Leitlinie für CPG empfiehlt [3]. Da das erstmalige Auftreten des Juckens und die Knotenbildungen der Haut der Hydroxychloroquin-Medikation und auch der Bosentan-Therapie vorangegangen waren, konnte eine medikamenteninduzierte CPG ausgeschlossen werden. Auch gab es des Weiteren keinen Hinweis auf eine andere zugrunde liegende Ursache. Da Myome am Uterus häufig vorkommen, ist die Diagnosestellung des Ovarialkarzinoms letztendlich der MRT-Empfehlung des Radiologen zu verdanken. Gemäß der gängigen wissenschaftlichen Literatur gibt es außer den malignen hämatologischen Erkrankungen nur wenige Fallbeschreibungen hinsichtlich des Auftretens von Karzinomen bei chronischem Pruritus oder CPG [1, 5]. Eine ursächliche Therapie für die CPG gibt es daher nur, wenn tatsächlich ein ursachenverdächtiger Befund gefunden wird. Daher werden gemäß Leitlinie symptomatisch topische und systemische Therapien empfohlen [3]. Diese helfen jedoch in vielen Fällen nur kurzfristig oder gar nicht, sodass der Bedarf an neuen Therapien groß ist. Erfreulicherweise wurden in der jüngsten Vergangenheit neue Wirkstoffe im Rahmen klinischer Studien, wie z. B. der κ‑Opioid-Rezeptor-Agonist Nalbuphin oder Nemolizumab, ein IL-31-Rezeptor-Antikörper, angeboten und deren Wirksamkeit bei CPG überprüft. Die ersten Publikationen demonstrieren eine gute Wirksamkeit bei CPG [4, 6].

In unserem dargestellten Fall hat die gründliche interdisziplinäre Durchuntersuchung bei neu aufgetretenem CPG richtungsweisende Befunde erbracht und eine maligne Erkrankung aufgedeckt. Der Beweis eines Ursachenzusammenhangs zwischen der CPG und dem Ovarialkarzinom ist nicht z. B. mit einem Test oder weiteren Untersuchungen zu erbringen, sondern kann nur über den klinischen und zeitlichen Verlauf mit Sistieren des Pruritus und der CPG wahrscheinlich gemacht werden. Bei unserer Patientin ging der Pruritus nach der Operation und Chemotherapie kontinuierlich zurück, und sie ist nun seit fast 6 Monaten pruritusfrei. Die CPG ist ebenso abgeheilt. Bei dieser Patientin handelt es sich um den – soweit uns bekannt – erstbeschriebenen Fall eines Ovarialkarzinoms bei CPG. Eine umfassende Diagnostik bei neu aufgetretener CPG ist daher stets angezeigt und wichtig, ganz besonders jedoch auch wegweisend für die weitere Behandlung. Dieser Fall demonstriert auch, dass eine gründliche Abklärung einer CPG ebenfalls lebensrettend sein kann.

## Fazit für die Praxis

- Eine plötzlich aufgetretene chronische Prurigo erfordert stets eine gründliche und interdisziplinäre Abklärung. Dabei sollte ganz besonders auf unklare Befunde eingegangen und diese weiter abgeklärt werden.
- Auch maligne Erkrankungen können im Zusammenhang mit einer CPG stehen und sollten bei der Ursachensuche berücksichtigt werden.
- Nemolizumab und Nalbuphin sind aktuell die hoffnungsvollsten neuen systemischen Wirkstoffe zur Therapie der CPG.
